# Tracheostomy tube change during the COVID-19 pandemic: timing and safety considerations

**DOI:** 10.1186/s43163-020-00032-2

**Published:** 2020-11-25

**Authors:** Mohamed Zahran, Ahmed Youssef

**Affiliations:** Department of Otolaryngology, Faculty of Medicine, Alexandria University School of Medicine, Champllion Street, El-Azareeta, Alexandria, Egypt

**Keywords:** Coronavirus, COVID-19, Tracheostomy, Personal protective equipment

## Abstract

**Background:**

The recent outbreak of SARS-CoV-2 has become pandemic since it began in late 2019. Tracheostomy is considered an aerosol-generating procedure which increases potential viral exposure to the health care staff.

**Main body:**

Since many patients diagnosed with SARS-CoV-2 underwent tracheostomy, the need for a standardized practice for tracheostomy tube change and care is mandatory. Articles listed in PubMed and guidelines from the otolaryngology societies were reviewed, and the state-of-the-art practice related to the topic is highlighted.

**Conclusions:**

Tracheostomy care in COVID-19 patients requires significant decision-making and preparation to carry out the task in a safe way and eliminate the risk of viral transmission.

## To the Editor

## Background

The recent outbreak of SARS-CoV-2 has become pandemic since it began in late 2019 [1]. Due to the high virulence via aerosol transmissions, to date, COVID-19 has infected more than 7 million people all over the world, causing more than 400,000 confirmed deaths [2]. Tracheostomy is an aerosol-generating procedure and increases potential viral transmission to the health care team [3]. As many COVID-19-positive patients underwent tracheostomy, the need for a standardized practice for tracheostomy tube change and care became mandatory. Reducing the risk of nosocomial infections through transmission of COVID-19 to other patients and health care workers is of critical importance [4].

## Main text

Articles listed in PubMed and guidelines from the AAO-HNS, ENT-UK, and French Society of Otolaryngology (SFORL) were reviewed, and the state-of-the-art practice related to the topic is highlighted.

### Ethical considerations

The Ethics Committee at the University Hospital approved this study, and informed consent was not mandatory.

The up to date information and practice guidelines are presented in the following sections.

### COVID-19 confirmed

#### Timing of tracheostomy cannula change

1. Both AAO-HNS and ENT-UK recommend no change of the tracheostomy tube until COVID-19 testing is negative and will have to review with infectious diseases.
2. Other authors recommend that the first tracheostomy change can be done after 7–10 days and subsequent change can be delayed 30 days after [5].

#### Technical considerations and safety precautions

##### Place of the procedure

Tracheal cannula management must be performed in a BSL-3 (biosafety level 3) [6] setting which is nowadays the standard for regular ward and ICUs (intensive care units) hosting COVID-19-positive patients [5].

##### Pre-procedure preparation

Surgical hand scrub must be done before and after each treatment. Dressing and undressing must be done within the room. The standard PPE (personal protective equipment) consists of an apron or a gown, head protection with a hood cap, N95 mask, protective glasses, and non-sterile gloves. The surgeon must ensure that all the necessary equipment are ready (correct size tracheostomy tube, suction catheters, lidocaine spray).

##### Details of the procedure

If the patient is on mechanical ventilator, anesthetist should sedate the patient and perform a muscle relaxation to minimize risk of coughing and aerosol generation. Five percent lidocaine is sprayed into the tracheostomy tube to anesthetize the airway and prevent coughing. A cuffed non-fenestrated tracheostomy tube should be used. Safe disposition of all equipment that came in contact with the patient must be done.

##### Nursing care

The tracheostomy care should be performed by trained nursing staff. The following is the state-of-the-art tracheostomy care:

- Suction should be done via closed circuit.
- Safe gentle suction and avoid unnecessary suctioning to reduce aerosolization.
- Cannula should be maintained appropriately inflated through regular checks of cuff pressure to avoid leak.
- Cannula should be held during any passive movement of the patient to avoid air leak from the stoma.
- Place HME (heat moisture exchange) with viral filter or a ventilator filter once the tracheotomy tube is disconnected from mechanical ventilation.
- Unnecessary dressing change should be avoided except if there is evidence of local infection [5].

These technical considerations in tracheostomy care are valid till there is documented evidence that the patient is recovered from COVID-19. The duration of contagiousness is still uncertain but is probably more than 25 days [7].

### COVID-19 unknown

#### The need to confirm COVID-19 status

Tracheostomized patients constitute significant infectious risk due to high aerosol generation. Preoperative COVID-19 testing is highly recommended prior to tracheostomy for all patients during the current pandemic. If tracheostomy was done without confirmation of COVID-19 status, postoperative COVID-19 testing should be done [8].

#### Precautions in unknown COVID-19 status

This situation is present when the patient is at home or outpatient clinic and no recent diagnostic test for COVID-19 is available. The following precautions are mandatory: N95 mask, protective glasses, gown, cap, or hood cap. All disposable equipment that came in contact with the tracheostomy tube or trachea (suction catheters) must be disposed appropriately. The tracheostomy tube should be connected to an HME filter and covered by a surgical mask to minimize risk of aerosol generation and droplet exposure [9].

## Discussion

Tracheostomy and tracheostomy care constitute a great risk for the medical staff due to aerosol generation. Several guidelines were recommended for the safety of the patients and caregivers. As many patients suffer from COVID-19 respiratory distress, the need for prolonged mechanical ventilation and tracheostomy care increases [1].

All the published guidelines recommend full PPE including N95 (USA), FFP2, or FFP3 (Europe) mask and the use of double gloves, goggles or eye protection, face shield, and an apron or gown [10–14]. The use of cuffed non-fenestrated tracheostomy tube was recommended by the American and Italian Otolaryngology guidelines. Additionally, both societies recommend that the cuff must be inflated all the time to avoid any air leak [10, 11]. The AAO-HNS and the South African Otolaryngology Society recommended the use of HME with viral filter to prevent viral transmission [10, 12]. The use of closed suction circuit was recommended by the American, South African, and British Societies [10, 12, 13].

There were controversies among guidelines in regard to the timing for the first tracheostomy tube change. The AAO-HNS and the South African Society did not recommend tube change until the patient became negative [10, 12]. In contrast, the Italian Society and ENT-UK suggested the first change to occur after 7–10 days [11, 13]. The Spanish guidelines advised to change tracheostomy tube after 14–21 days regardless of the COVID-19 status [14].

## Conclusions

Although tracheostomy care was previously considered routine, it poses significant risk of viral exposure to health care staff and the community. The presented state-of-the-art practice is a guide for health practitioners involved in COVID-19 patients’ care. Further evidence-based guidelines are needed in the near future as our understanding of this novel disease is evolving.

## Data Availability

Not applicable

## Abbreviations

AAO-HNS: American Academy of Otolaryngology**-**Head and Neck Surgery
BSL: Biosafety level
COVID-19: Coronavirus disease
HME: Heat moisture exchanger
ICU: Intensive care unit
PPE: Personal protective equipment
SARS-CoV-2: Severe acute respiratory syndrome caused by coronavirus 2
